# Balancing Between Privacy and Utility for Affect Recognition Using Multitask Learning in Differential Privacy–Added Federated Learning Settings: Quantitative Study

**DOI:** 10.2196/60003

**Published:** 2024-12-23

**Authors:** Mohamed Benouis, Elisabeth Andre, Yekta Said Can

**Affiliations:** 1 Faculty of Applied Computer Science, Augsburg University, Augsburg, Germany

**Keywords:** privacy preservation, multitask learning, federated learning, privacy, physiological signals, affective computing, wearable sensors, sensitive data, empathetic sensors, data privacy, digital mental health, wearables, ethics, emotional well-being

## Abstract

**Background:**

The rise of wearable sensors marks a significant development in the era of affective computing. Their popularity is continuously increasing, and they have the potential to improve our understanding of human stress. A fundamental aspect within this domain is the ability to recognize perceived stress through these unobtrusive devices.

**Objective:**

This study aims to enhance the performance of emotion recognition using multitask learning (MTL), a technique extensively explored across various machine learning tasks, including affective computing. By leveraging the shared information among related tasks, we seek to augment the accuracy of emotion recognition while confronting the privacy threats inherent in the physiological data captured by these sensors.

**Methods:**

To address the privacy concerns associated with the sensitive data collected by wearable sensors, we proposed a novel framework that integrates differential privacy and federated learning approaches with MTL. This framework was designed to efficiently identify mental stress while preserving private identity information. Through this approach, we aimed to enhance the performance of emotion recognition tasks while preserving user privacy.

**Results:**

Comprehensive evaluations of our framework were conducted using 2 prominent public datasets. The results demonstrate a significant improvement in emotion recognition accuracy, achieving a rate of 90%. Furthermore, our approach effectively mitigates privacy risks, as evidenced by limiting reidentification accuracies to 47%.

**Conclusions:**

This study presents a promising approach to advancing emotion recognition capabilities while addressing privacy concerns in the context of empathetic sensors. By integrating MTL with differential privacy and federated learning, we have demonstrated the potential to achieve high levels of accuracy in emotion recognition while ensuring the protection of user privacy. This research contributes to the ongoing efforts to use affective computing in a privacy-aware and ethical manner.

## Introduction

### Background

Imagine awakening in the morning to find yourself dealing with stress, perhaps stemming from a disagreement with a friend, a pressing financial concern, or an unresolved work–related matter. In this scenario, an advanced digital assistant, akin to Siri or Alexa, seamlessly detects the user’s stress level through the analysis of physiological signals captured by wearable technology. Leveraging this data, the digital assistant dynamically adjusts its language and tone to suit the user’s mood. Moreover, it proactively recommends personalized relaxation techniques, drawing from past successful interventions such as yoga, mindfulness practices, listening to favorite music, and watching uplifting videos. Such a sophisticated stress recognition and intervention system holds promise for enhancing the well-being and emotional resilience of individuals. Although most of the studies use facial expressions [1] and speech [2] for recognizing stress and emotions (ie, affects as an umbrella term), physiology-based methodologies also emerged as an alternative since they can offer promise for seamless, continuous monitoring within everyday contexts. Notably, 330 million smartwatches, fitness trackers, and wearables were sold in 2020, which are capable of gathering quantitative physiological data [3]. They are promising tools for affect recognition due to their unobtrusive data collection capabilities.

Multitask learning (MTL) is a method proposed to tackle training multiple related tasks at the same time. It works by sharing knowledge between these tasks to make each model perform better [4]. Essentially, MTL acts like a behind-the-scenes helper, enhancing the ability of machine learning (ML) models to generalize different types of data [5]. This technique has been particularly useful in improving ML models across various fields, including affective computing [6]. For instance, if we want to improve how a system recognizes emotions, we can also train it to recognize gender [7]. By doing this, the system can learn from both tasks simultaneously, making it more effective in recognizing emotions and enhancing performance. The motivation for using identity and emotion has also a psychological basis. Connolly et al [8] examined whether facial responses are shared between identity and emotion. They showed that there is a strong positive correlation between face emotion recognition and face identity recognition tasks, and the 2 recognition tasks share a common processing mechanism. Sheng and Li [9] also confirmed the dependence of identity and emotion tasks by using a gait signal. Inspired by the MTL idea and previous studies, we used MTL to perform each task separately, stress recognition and identity recognition, respectively, by using physiological signals. However, there is a concern when third parties gain access to additional information, like gender, identity, or age, which could be misused for targeted advertising or even discrimination in things like job opportunities [10]. Moreover, if sensitive information like biometric data gets leaked, it could lead to cybercrimes such as identity theft. Therefore, it is crucial for MTL systems to incorporate privacy-preserving methods.

Researchers have introduced federated learning (FL) as a way to address privacy issues. FL allows users to train their data locally while sharing only the trained model parameters rather than the original data itself. The philosophy behind FL is to bring code to the data instead of moving the data to the code [11]. This method complies with privacy regulations such as the European Union’s General Data Protection Regulation [12] and the California Consumer Privacy Act [13]. FL has shown promise in safeguarding user privacy in Internet of Things networks.

Although FL is a significant step forward in protecting user privacy, it is not invincible. There is still a risk of sensitive information being uncovered by reverse engineering the local model parameters. To further enhance privacy, researchers have integrated privacy-preserving techniques into ML for physiological data. One such method is differential privacy (DP), which adds random noise to each model in the client or server, disrupting updates and limiting the leakage of sensitive information between nodes [14]. However, it is worth noting that DP can reduce the performance of ML models.

In practice, protecting the privacy of users without degradation in model utility is still an open problem. In this study, we first implemented an MTL architecture for recognizing stress and identity from multimodal physiological signals. We further added FL and DP mechanisms to preserve privacy. To obtain a robust performance, we separated the 2 tasks and added noise to only the privacy task, which is biometric identity recognition. In this way, we were able to improve the stress recognition performance with the help of MTL and hide identity information by adding noise to the identity task model by using DP. To the best of our knowledge, this study is the first MTL-based affect recognition study using FL and DP to preserve privacy at the same time.

In the next section, we mentioned the related works using MTL and FL for affect recognition. We then presented our approach in the *Methods* section. In the *Results* section, we compared multitask centralized, decentralized FL, and decentralized FL with DP approaches on selected public datasets for recognizing stress levels and identity of users. We concluded the study with the lessons learned, limitations, and future works in the *Discussion* section.

### Related Works

To develop a robust affective computing system that can be used in practical applications, researchers tested various modalities with state-of-the-art deep learning techniques. MTL has also raised significant attention from various domains over the past few years, including affective computing. Chen et al [14] applied it to audiovisual signals in the Audio/Visual Emotion Challenge and achieved a significant performance increase compared to baseline ML algorithms by detecting arousal and valence levels simultaneously. Since the human face source can be used for several tasks, such as gender recognition or age estimation, Sang et al [7] used MTL with convolutional neural network (CNN) for smile detection, emotion recognition, and gender classification.

They outperformed the state-of-the-art techniques in selected 3 benchmark datasets (Internet Movie Database and Wiki dataset, GENKI-4K dataset [a dataset containing images labeled with facial expressions, commonly used in emotion recognition research], and Facial Expression Recognition Challenge-2013 dataset). MTL-based centralized learning (CL) for affective computing is beneficial for simultaneously sharing features and learning auxiliary tasks. However, when private tasks (ie, face, gender, and person detection) are included in MTL to improve affect recognition performances, the models create the risk to reveal this sensitive information to possibly malicious parties especially while uploading the features to the central server for both utility and auxiliary tasks for learning.

Data privacy has become an issue of great concern in affect recognition using either verbal or nonverbal data, as gender, age, and identity of the user can be revealed in the process. FL is proposed to preserve privacy while taking advantage of ML and has attracted significant attention from various domains over the past few years, but affective computing research and applications on emotion-related tasks are rarely discussed. Most existing works are conducted on private datasets or in limited scenarios, making it difficult for researchers to compare their methods fairly [15].

FL has been widely applied for facial features for affect recognition. Somandepalli et al [16] investigated FL for 2 affective computing tasks: classifying self-reports and perception ratings in audio, video, and text datasets. Using the speech modality, Latif et al [17] investigated an FL approach in emotion recognition tasks while sharing only the model among clients. They implemented a long short-term memory classifier and tested it on the interactive emotional dyadic motion capture dataset with 4 emotions: happy, sad, angry, and neutral. Face and speech modalities were also often combined to get a more robust performance [18]. Chhikara et al [19] combined emotion recognition from facial expressions and speech with an FL approach. For the face modality, they used a combination of CNN and support vector machine models, whereas for the audio modality, they applied a 2D CNN model to extract spectrogram images. Their proposed framework has been validated and tested on 2 datasets, Facial Expression Recognition 2013 for facial emotion recognition and Ryerson Audio-Visual Database of Emotional Speech and Song for speech emotion recognition, respectively.

On the contrary, FL has been seldom used in the context of affect recognition from physiological signals, as indicated in Table 1. Can and Ersoy [15] implemented an FL learning model to forecast perceived stress using physiological data. Each subclient uses an multilayer perceptron classifier to locally train its data on the edge, and the sharing of individual updating parameters of multilayer perceptron is facilitated using the federated averaging (FedAvg) algorithm. FL has also been extended to handle multimodal physiological signals. Nandi and Xhafa [20] proposed Federated Representation Learning for Multi-Modal Emotion Classification System, an FL framework-based ML model for emotion recognition. They applied wavelet feature extraction and a neural network to electrodermal activity (EDA) and respiration data from the Dataset for Emotion Analysis using Physiological signals to recognize valence arousal levels, validating their approach. These studies demonstrate the successful application of FL without compromising affect recognition performance.

**Table 1.** Studies using MTL^a^ or privacy-preserving approaches for various applications.

Study	Application	Signal	MTL	FL^b^	DP^c^	Multimodality
Zhao et al [21]	Network anomaly detection	Network signals	✓	✓		
Smith et al [22]	Activity recognition	Acceleration, gyroscope	✓	✓		✓
Somandepalli et al [16]	Affect recognition	Speech, video, text		✓		✓
Sang et al [7]	Affect recognition	Face images	✓			
Shome and Kar [23]	Affect recognition	Face images		✓		
Chhikara et al [19]	Affect recognition	Face and speech		✓		✓
Feng et al [24]	Affect recognition	Speech		✓	✓	
Can and Ersoy [15]	Affect recognition	PPG^d^		✓		
Nandi and Xhafa [20]	Affect recognition	EEG^e^, EDA^f^		✓		✓
Wang et al [18]	Biometric	Face speaker		✓	✓	✓
This study	Affect recognition	PPG, EDA, ACC^g^, ST^h^	✓	✓	✓	✓

a MTL: multitask learning.

b FL: federated learning.

c DP: differential privacy.

d PPG: photoplethysmography.

e EEG: electroencephalography.

f EDA: electrodermal activity.

g ACC: acceleration.

h ST: skin temperature.

While FL has been introduced to enhance model training in terms of privacy, the privacy vulnerabilities inherent in the stochastic gradient descent (SGD) algorithm remain unresolved. DP mechanisms have primarily been discussed in FL settings, involving the injection of noise into each model client or server to perturb updates and limit gradient leakage shared among nodes (ie, client and server) [25]. In one of the initial applications, authors introduced a new private training method termed differential private SGD, which reduces local and global gradient information leakage between the client and server. Instead of using the standard composition theorem to calculate the final distribution of overall noise clients, they used a moments accountant metric to adaptively monitor the overall privacy loss. Recognizing that servers are often curious or untrustworthy, Wei et al [26] proposed a local DP mechanism algorithm by introducing Gaussian noise distribution into user models before uploading them to servers. To address the communication overhead required for optimal convergence upper bound for DP, they introduced a novel approach known as communication rounds discounting method, which achieves a better trade-off between the computational complexity of searching and convergence performance. DP has also been leveraged for affect recognition. Feng et al [24] used user-level DP to mitigate privacy leaks in FL for speech emotion recognition. Recently, Smith et al [22] showcased promising performance in preserving privacy via multitask federated learning (MFL) for activity recognition.

When examining the affective computing literature, we find studies using MTL to improve performance and others using FL or DP to preserve privacy. However, there are no studies that leverage MTL for performance enhancement while also preserving privacy in this literature. The overall idea of our approach is to separate the utility and the privacy tasks preventing DP from compromising the performance of the utility task (affect recognition) and introducing noise exclusively to the privacy task (biometric identity recognition). Consequently, our work represents the first MTL-based approach to affect recognition using FL and DP to preserve privacy at the same time.

## Methods

### Overview

**Figure 1.**
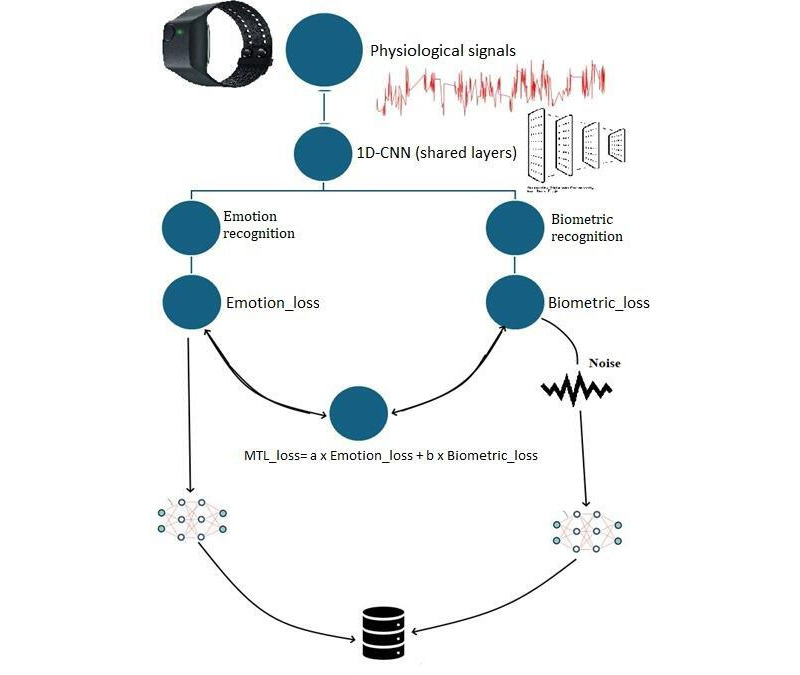
Block diagram of the proposed MTL system. CNN: convolutional neural network; MTL: multitask learning.

Our proposed framework is divided into 3 main substeps: feature extraction, FL model, and FL with DP settings (see Figure 1 for the block diagram).

### Ethical Considerations

This study did not require formal ethics approval, as it exclusively used publicly available datasets, which were obtained from open-access repositories with no personally identifiable information. According to the General Data Protection Regulation, studies involving the use of anonymized, publicly accessible data that do not engage human participants directly are exempt from ethics review. Therefore, no ethics approval was sought or required.

### Data Description and Feature Extraction

For our experiments, we use 2 datasets, wearable stress and affect detection (WESAD) [27] and virtual environment for real-time biometric interaction and observation (VERBIO) [28], which have been created for affective state monitoring.

#### WESAD Dataset

In the WESAD dataset, each participant recorded physiological signals such as blood volume pulse, electrocardiogram (ECG), EDA, electromyogram, respiration, body temperature, and 3-axis acceleration measured from the chest and wrist using Plux RespiBAN and Empatica E4 devices. In total, 15 (12 male and 3 female) people participated in this experience. There were 4 states: baseline, amusement, stress, and meditation. More details can be found in Schmidt et al [27].

#### VERBIO Dataset

The VERBIO dataset [28] consists of biobehavioral responses and self-reported data during public speaking presentations, both in front of real-life and virtual audiences. In total, 55 participants conducted 10 distinct presentations, each lasting approximately 5 minutes, across the 3 segments of the study, spanning 4 days: PRE (1 session, day 1), TEST (8 sessions, days 2‐3), and POST (1 session, day 4). The PRE and POST segments involved real-life audiences, while the TEST segment featured various virtual audiences. To prevent participant exhaustion, the TEST segment was divided into 2 days, each comprising 4 sessions. In total, the study yielded 10,800 minutes of acoustic and physiological data from 82 real and 216 virtual reality presentations. In total, 35,174 segments were created. We solely used physiological data collected using the Empatica E4 device, possessing identical sampling rates and modalities as those used in the WESAD dataset.

#### Preprocessing

Each modality of the signal is segmented using 700 sample windows size with 50% overlap, as suggested in the literature [29]. To maximize the correlation among interparticipants and minimize among participants, these segments were further processed for extracting features [15] such as mean, variance, root mean square, frequency domain features, average first amplitude difference, second amplitude difference, skewness, kurtosis, and entropy as a nonlinear feature.

### Decentralized MFL Model

For privatizing the user identity while preserving stress recognition accuracy, we adopted an MFL approach that can effectively improve the performance of stress recognition while limiting the risk of inferring sensitive information from the training model since the client does not want to be exposed to the cloud service provider. MFL architecture-based stress recognition is developed as follows:

1. The dataset is partitioned into *K* clients. The data size of all the clients is the same. The client distribution is also assumed as independent and identically distributed (IID) and no IID [10].^10^
2. For the local training process, there is only 1 iteration for SGD local training for each client. In particular, **w** is the local model parameter [10] given by:

(1) $$w_{U}^{D_{i}}=arg\underset{w_{U}}{min}\left(F_{U}\left(w_{U}\right)+\frac{\mu }{2}{\left\|w_{U}-w^{\left(D_{i}-1\right)}\right\|}^{2}\right)$$

where μ is the learning rate and $F_{U}$ is the local loss function of the *k*th user.

1. The local data of different clients cannot be communicated, and only the models can be shared.^10^,^10^
2. Following the FedAvg algorithm [10], the server uses a global averaging approach to aggregate all local training models to compute the final global model. Formally [10], the server aggregates the weights sent from the *K* clients as follows:

(2) $$w=\sum_{U=1}^{K}‍p_{i}w_{U}^{D_{i}}$$

where $w_{i}$ is the parameter vector trained at the *k*th client, w is the parameter vector after aggregating at the server, K is the number of participating clients, $D_{i}$ is the dataset size of each participating client, $D=UD_{i}$ is the whole distributed dataset, and *P_i_*=|*D_i_*|/|*D*|.

1. The global training epoch is set to M rounds (aggregations). The server solves the optimization problem [10]:

(3) $$W^{*}=arg\underset{w_{U}}{min}\sum_{U=1}^{M}‍P_{U}F_{U}\left(w_{U},D_{i}\right)$$

where $F_{U}$ is the local loss function of the *k*th client. Generally, the local loss function is given by local empirical risks.

### Decentralized FL With DP

In conventional FL, the global model is computed through averaging over model client participants, which performs better within homogeneous FL settings. However, using inference or adversarial attack, this shared model may contain sensitive and private information such as gender, age, and biometric template user. In such cases, the MFL framework is required to reduce the leakage of the black box gradient exchanged model. To overcome this limitation, researchers have used the DP scheme to protect either local or global data training FL model. However, the perturbed gradient using DP with a low budget has high variance, which leads to worse performance and slower convergence. We also compared adding noise to whole model and task-specific last layers in addition to the shared layers and demonstrated the performance of these 3 different approaches (Multimedia Appendix 1). Motivated by personalized FL [26], our work focuses on client-level privacy, which aims at a private specific layer of the client model rather than perturbing the entire whole local model. This is because the base layers are mostly redundant information, while the most important information that holds private and public information is located in the upper layers. To meet the utility privacy trade-off, the DP mechanism is used to perturb the gradients using Gaussian noise at a specific layer or task. Here, we use all steps in the FL model except step 4, that is, before uploading the local SGD model client to the global server, we inject an amount of noise to the updated local parameters. In that sense, we perturb the local gradient training inference with two kinds of noise distributions:

1. An additive Gaussian noise *η*∼*N* (0, *σ*) to each weight. This operation can be mathematically described as follows:

(4) $$w_{t+1}=w_{t}+\eta$$

1. A set of noise distributions can be sampled from the DP mechanism. A randomized mechanism M on the training set with domain *X* and range *R* satisfies (*ϵ*, *δ*) − DP for 2 small positive numbers and if the following inequality holds [25]:

(5) $$Pr[M(x)\in S]\leq e^{\epsilon }Pr\left[M\left(x^{`}\right)\in S\right]+\delta$$

where x and $x^{`}$∈X are 2 input neighbor datasets, and S⊆R, where R is the set of all possible outputs, δ is privacy loss or failure probability, and ϵ is privacy budget.

An ideal DP mechanism provides a lower value of δ and a smaller value of ϵ. Unfortunately, these values decrease the function utility (eg, accuracy metric), so the main question is how much DP values we must perturb its output while guaranteeing trade-off privacy-utility. Intuitively, an output perturbation mechanism takes an input x and returns a random variable s(x). This operation can be modeled by:


(6) $$M\left(x\right)=s\left(x\right)+N_{\sigma }$$

where N is scaling noise sampled from a specific distribution. In this work, we chose Laplace and Gaussian mechanisms [25] that use L1 and L2 norm sensitivity, respectively. The sensitivity function can be expressed as:


(7) $$\Delta f=\underset{D,D^{`}}{max}\left\|s(x)-s{\left(x^{`}\right)}_{1,2}\right\|$$

Scaling noise can be computed as:


(8) σ=Δf/ε

Output perturbation satisfies (ϵ,δ)-DP when we properly select the value scaling noise. Thus, it sampled from Laplace and Gaussian distributions [25] as:


(9) $$M_{Laplace}\left(x,f,\varepsilon ,\delta \right)=s\left(x\right)+Lap\left(\mu =0,b\right)$$

(10) $$M_{Gaussian}(x,f,\varepsilon ,\delta )=f(x)+N\left(\mu =0,\sigma^{2}\right)$$

The gradient information leakage can be reduced by applying a gradient thresholding or clipping algorithm. As explained by Abadi et al [11], gradient clipping is crucial in ensuring the DP of FL algorithms. So, each provider or client model update needs to have a bounded norm, which is ensured by applying an operation that shrinks individual model updates when their norm exceeds a given threshold. Clipping impacts of an FL algorithm’s convergence performance should be known to create FL algorithms that protect DP.

## Results

### Overview

Three scenarios are created to tackle the aforementioned challenges with the DP learning approaches: centralized, decentralized FL, and decentralized FL with DP. Their performances are evaluated on WESAD and VERBIO datasets on 2 different tasks. The first task is identifying users from a set of registered and recorded users. The second task is perceived binary stress recognition, which tries to distinguish the user’s stress level, stress versus nonstress. We train a multitask deep learning model for handling these tasks simultaneously. In addition, for better training and to avoid overfitting, an early stopping regularization technique is used as gradient descent. The accuracy metric is used for measuring identification and stress recognition performance. In each simulation scenario, we run 5-fold cross-validation, where each fold is tested based on the training of the other 4. Inspired from a successful architecture [30] and as described in Table 2, the multitask 1D-CNN model is based on 3 convolutional layers, a pooling layer, 2 fully connected layers, and 2 linear classifiers to classify the studied tasks. The multitask model uses the cross-entropy loss function and SGD learning rate (β=.0005).

**Table 2.** Hyperparameters of the multitask 1D-convolutional neural network–based architecture.

Layer	Type	Hyperparameters
Input	Input	Features size
Conv1D	Convolution	Input=1, output=20, *K*=8, stride=1, padding
ReLu	Activation function	ReLu
Pooling	Pooling	Stride=2, max pooling
Conv	Convolution layer	Input=20, output=40, *K*=8, stride=1, padding
Relu	Activation function	ReLu
Pooling	Pooling	Stride=2, max pooling
Conv	Convolution layer	Input=40, output=60, *K*=8, stride=1, padding
Relu	Activation function	ReLu
Pooling	Pooling	Stride=2, max pooling
Fully connected 1	Fully connected layer	Input=360, output=100
Fully connected 2	Fully connected layer	Input=100, output=300
Linear 1	Input: 100, output: 2	Output=2 affect classes, activation function:linear
Linear 2	Input: 300, output: N	Output=N identity classes, activation function:linear

For each target task, the individual loss is determined by the cross-entropy for both stress recognition (Loss_1_) and identification tasks (Loss_2_). The individual losses are summed and form the total cost function (Loss_T_).

### CL Approach

Most existing stress recognition studies in conventional centralized ML settings use WESAD to position their works against state of the art [31-33] and achieve over 95% accuracy for binary stress recognition. Similarly, we used the CL approach on the WESAD dataset as a baseline experiment. We measured the performance of stress levels for each participant independently and then computed the average performance accuracy. Each individual participant model is trained using 5-fold cross-validation.

Multimedia Appendix 2 shows the results of our CL approach using a 1D-CNN multitask model for stress and identity recognition tasks on 2 datasets: WESAD and VERBIO. After training, the output layers are used to infer the stress level and the identity of the user, resulting in quite similar average scores of 99%. The results reveal a potential information leakage in this case, wherein model accuracy is preserved at the expense of the user’s privacy. As a result, this approach fails to ensure the users’ privacy since their data are transmitted to the server for training purposes.

### Multitask Decentralized FL Approach (MFL)

Here, *K* (ie, participating clients) training models were created to train the whole dataset and the size of local samples *D_i_*=1000. We set the number of training epochs (communication round) to *T*=40 and local training epochs to 1. Overall, the average accuracy result achieved is 97% and 95% for stress mood recognition and 93% and 90% for user identification on WESAD and VERBIO, respectively.

**Figure 2.**
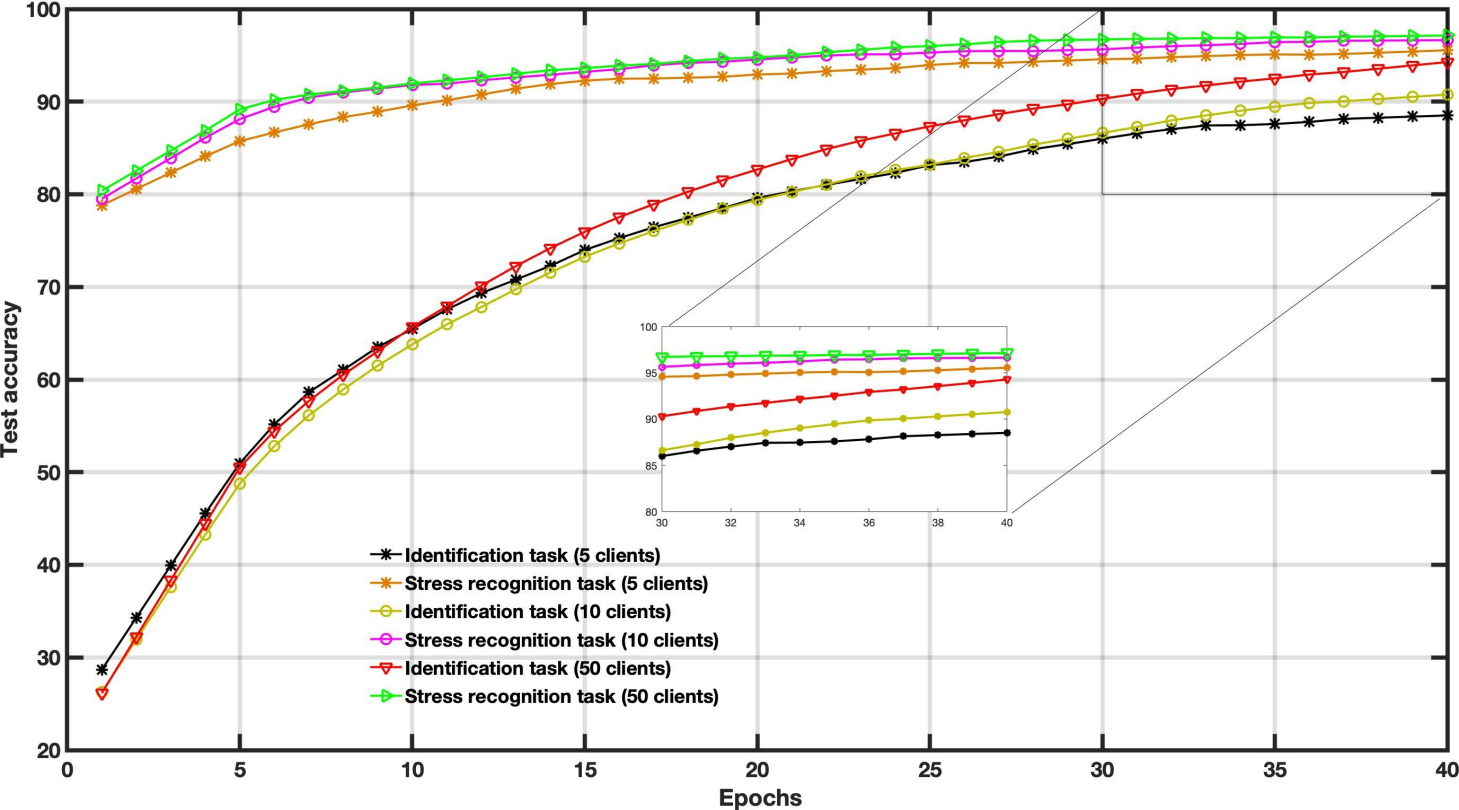
The impact of the user participant size (various number of clients) on the multitask federated learning performance (wearable stress and affect detection).

**Figure 3.**
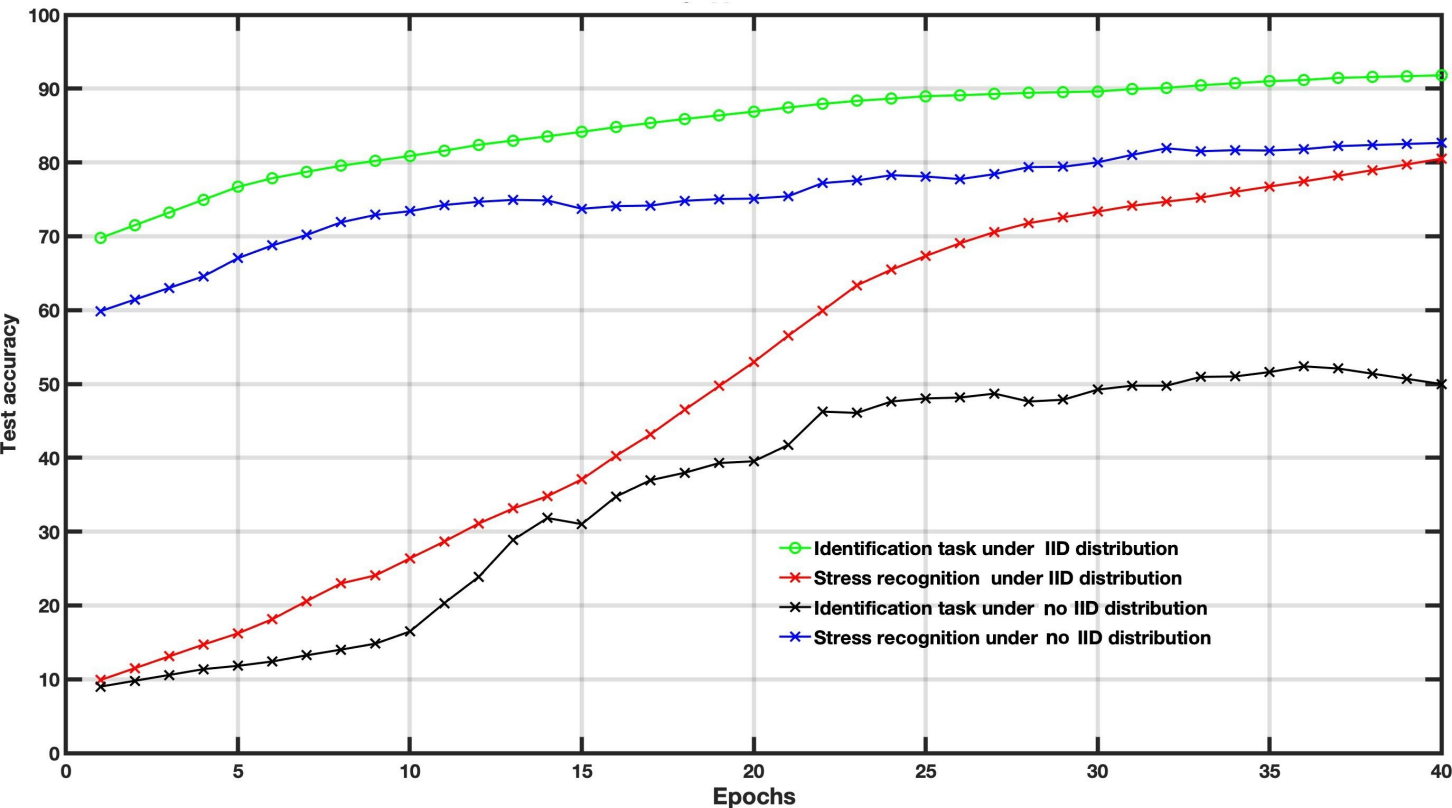
The impact of IID versus no IID distribution on the multitask federated learning performance. IID: independent and identically distributed.

To examine the effect of client participation within the MFL model, we tested different numbers of clients, that is, *K*=5, *K*=10, and *K*=20. As reported by Wang et al [34] and confirmed in Figure 2, an increasing number of clients and more client participation provide better performance for MFL training. The client distribution is different in assessing the MFL model in real-world conditions. We compare the convergence performance of the MFL model under IID and no IID for both tasks: stress recognition and subject identification (Figure 3). We note that the data distribution dramatically affects the quality of the FL training and obviously affects MFL’s convergence performance. IID is a more idealistic case, whereas no IID is a more realistic case. Therefore, we expect the result to be lower in no IID cases. The performance difference proves the importance of data distribution.

Adjusting FL hyperparameter setting results can achieve a better performance than the CL approach. However, it may lead to a lower privacy level. As a result, SGD training may still reveal sensitive information about the client while exchanging the ML model with the global server.

### MFL With DP Approach

1. To highlight the benefits of our proposed approach, we examine the impact of injecting noise into the local client training network according to these 3 scenarios: the full layers, shared layers, and task-specific layers (Figure 2). The used noise is sampled via the following mechanisms:
2. With DP technique–based Laplace and Gaussian mechanisms, the noise scale is drawn from the output perturbation mechanism. DP parameters are computed at each local training round to generate appropriate noise injected from specific distributions (ie, Laplace and Gaussian). Besides appropriate (ϵ,δ)−DP initialization, there are a few hyperparameters to be tuned, such as the number of clients N, the number of maximum communication rounds *T*, and the number of chosen clients K.
  - Laplace distribution [11] is computed as:
(11) $$\sigma =\frac{\Delta f}{\epsilon }$$
  - Gaussian distribution—2 distributions are given by Abadi et al [11] and Schmidt et al [27], respectively:
(12) $$\sigma_{1}=\sqrt{\frac{2log\frac{1.25}{\delta }}{\varepsilon }}$$
(13) $$\sigma_{2}=\frac{\Delta f\sqrt{2qTlog\left(\frac{1}{\delta }\right)}}{\varepsilon }$$

 where $\Delta f=\frac{2C}{U_{i}}$, $q=\frac{K}{N}$, and C = clipping threshold. We set the clipping factor to 1 and δ to 0.00001.

**Figure 4.**
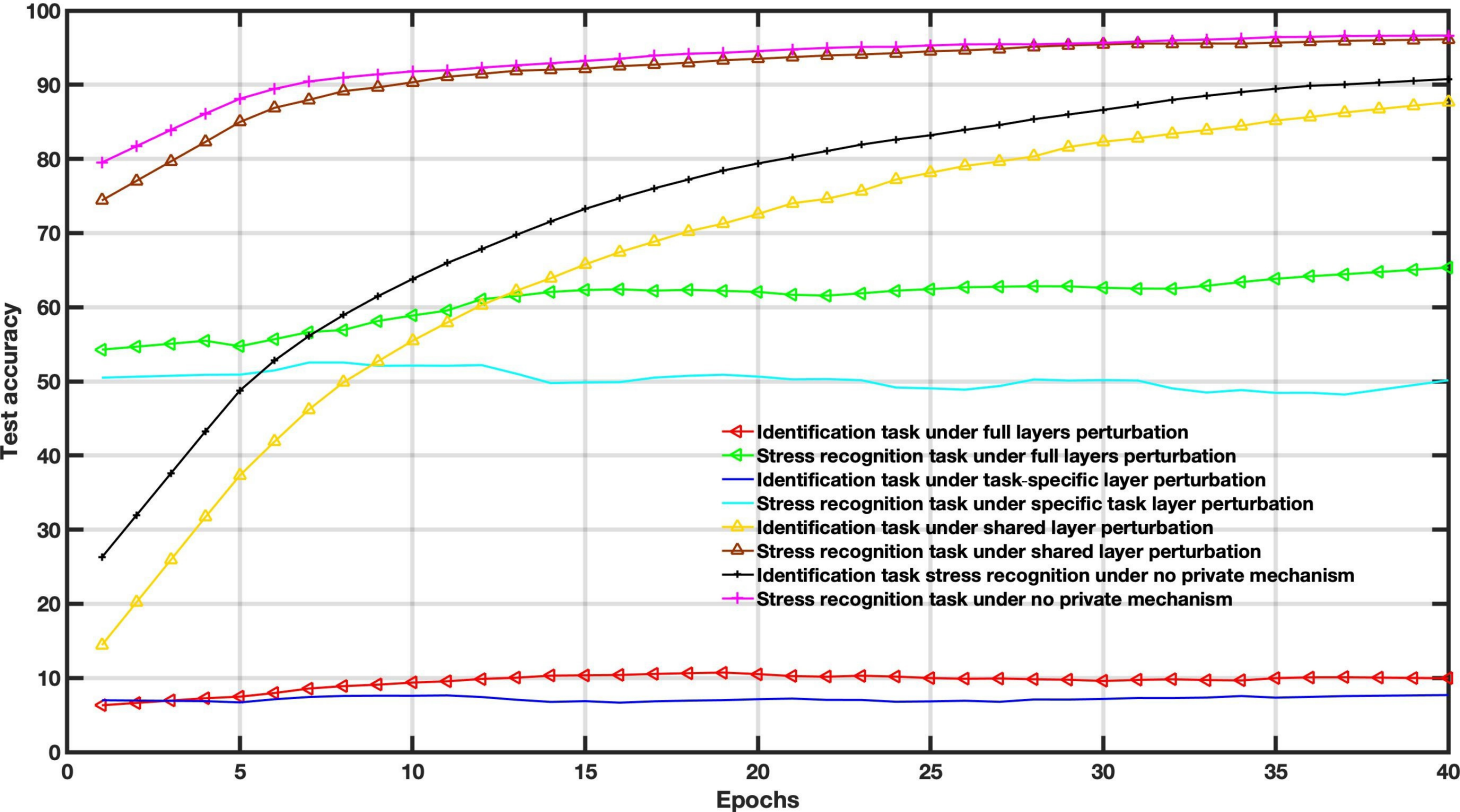
Decentralized multitask federated learning approach results under different Gaussian noise levels (*σ*=0.1).

**Figure 5.**
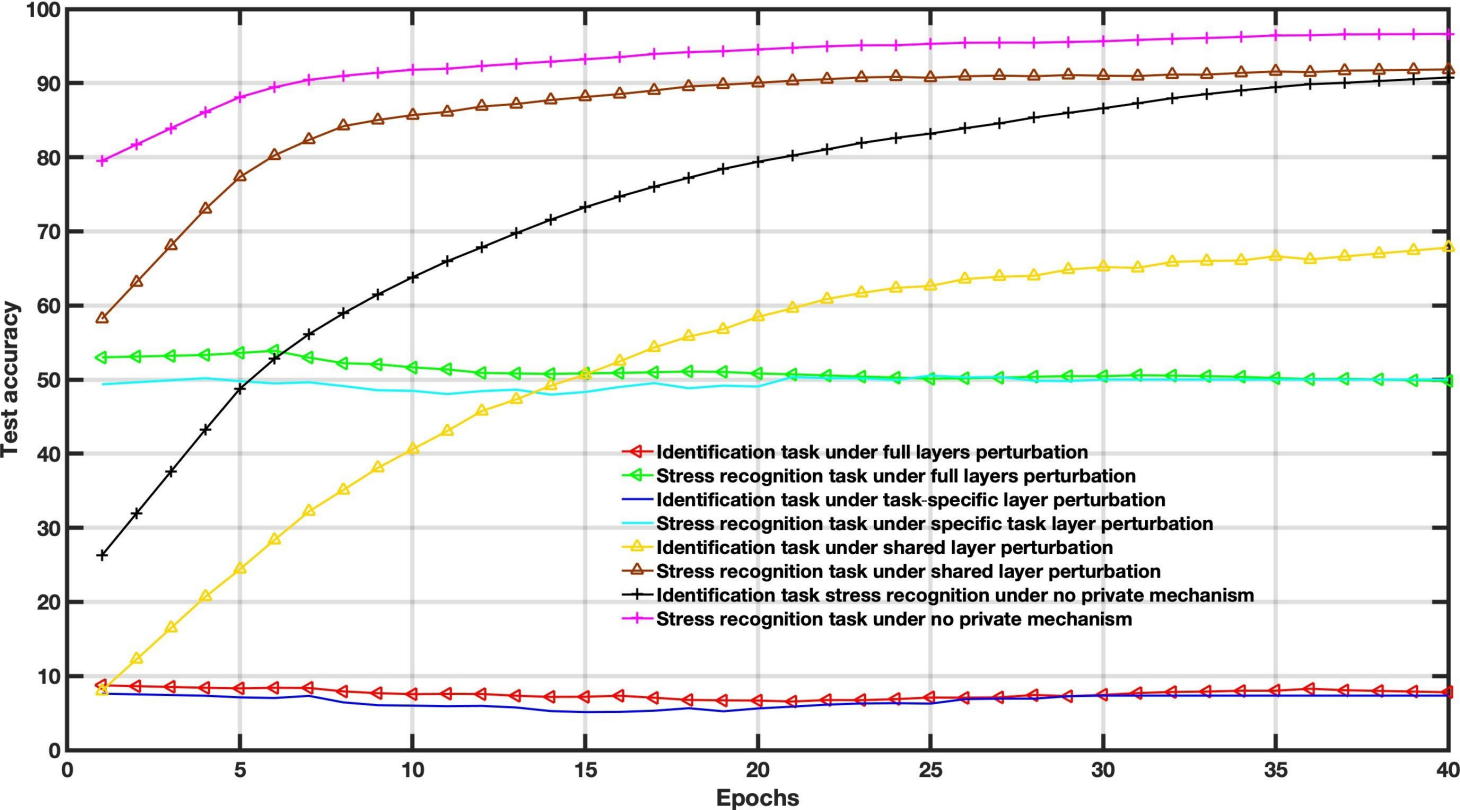
Decentralized multitask federated learning approach results under different Gaussian noise levels (*σ*=0.3).

**Figure 6.**
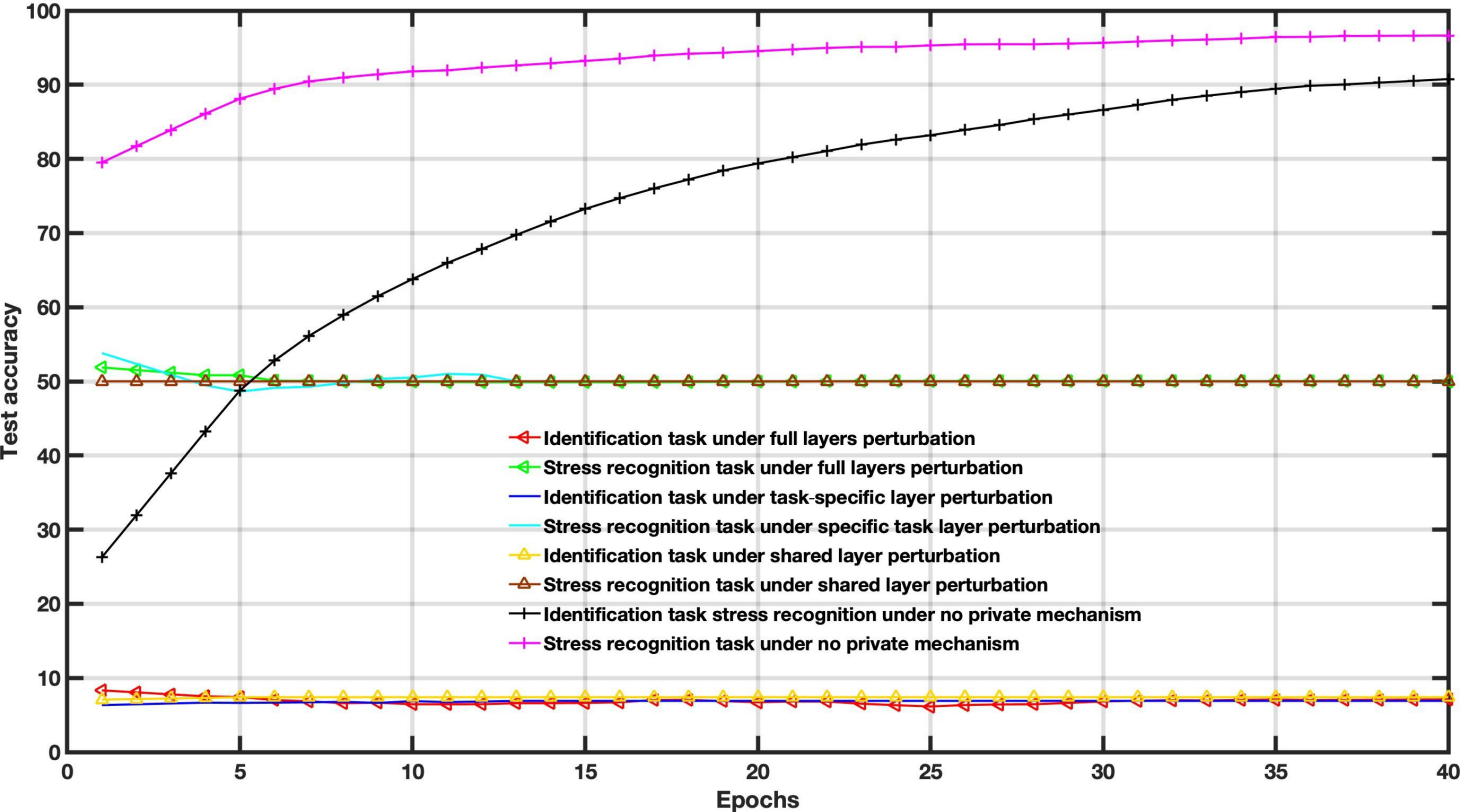
Decentralized multitask federated learning approach results under different Gaussian noise levels (*σ*=0.6).

Figures 4-6 show the accuracy comparison when adding Gaussian distribution levels into local training according to the 3 scenarios on the WESAD dataset. Compared to the baseline scenario, that is, no private mechanism, the perturbing share layer scheme with σ=0.1 and σ=0.3 only provides better results for utility tasks; however, the identification task reached an accuracy of around 86%.

In this case, the amount of noise drawn from the Gaussian distribution is used to balance the utility and privacy and does not consider the FL settings parameters. The Gaussian levels are set to *σ*=0.1, *σ*=0.3, and *σ*=0.6. For instance, increasing Gaussian noise leads to poor performance, as depicted in Figure 6.

**Figure 7.**
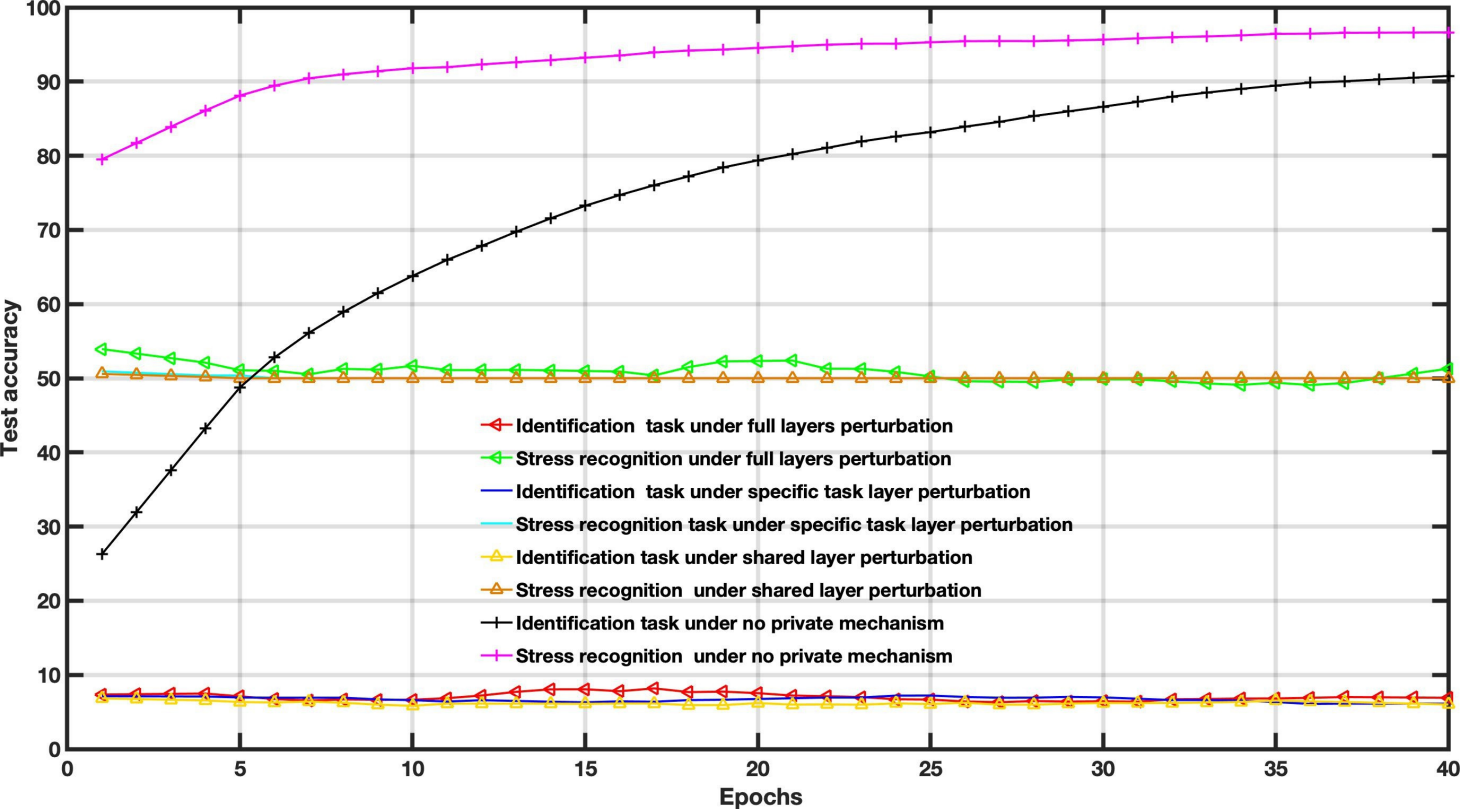
Multitask federated learning performance under the differential privacy mechanism “different layer perturbations” (wearable stress and affect detection, *ϵ*=1).

**Figure 8.**
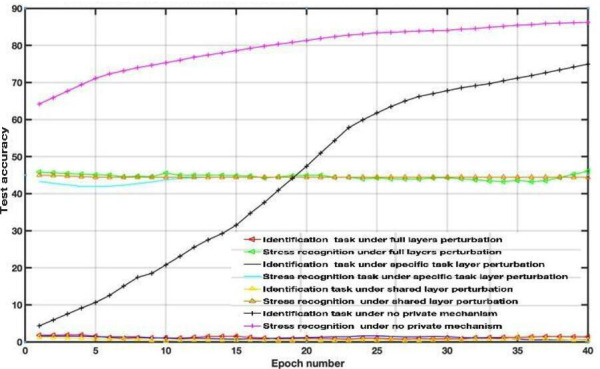
Multitask federated learning performance under the differential privacy mechanism “different layer perturbations” (virtual environment for real-time biometric interaction and observation, *ϵ*=1).

**Figure 9.**
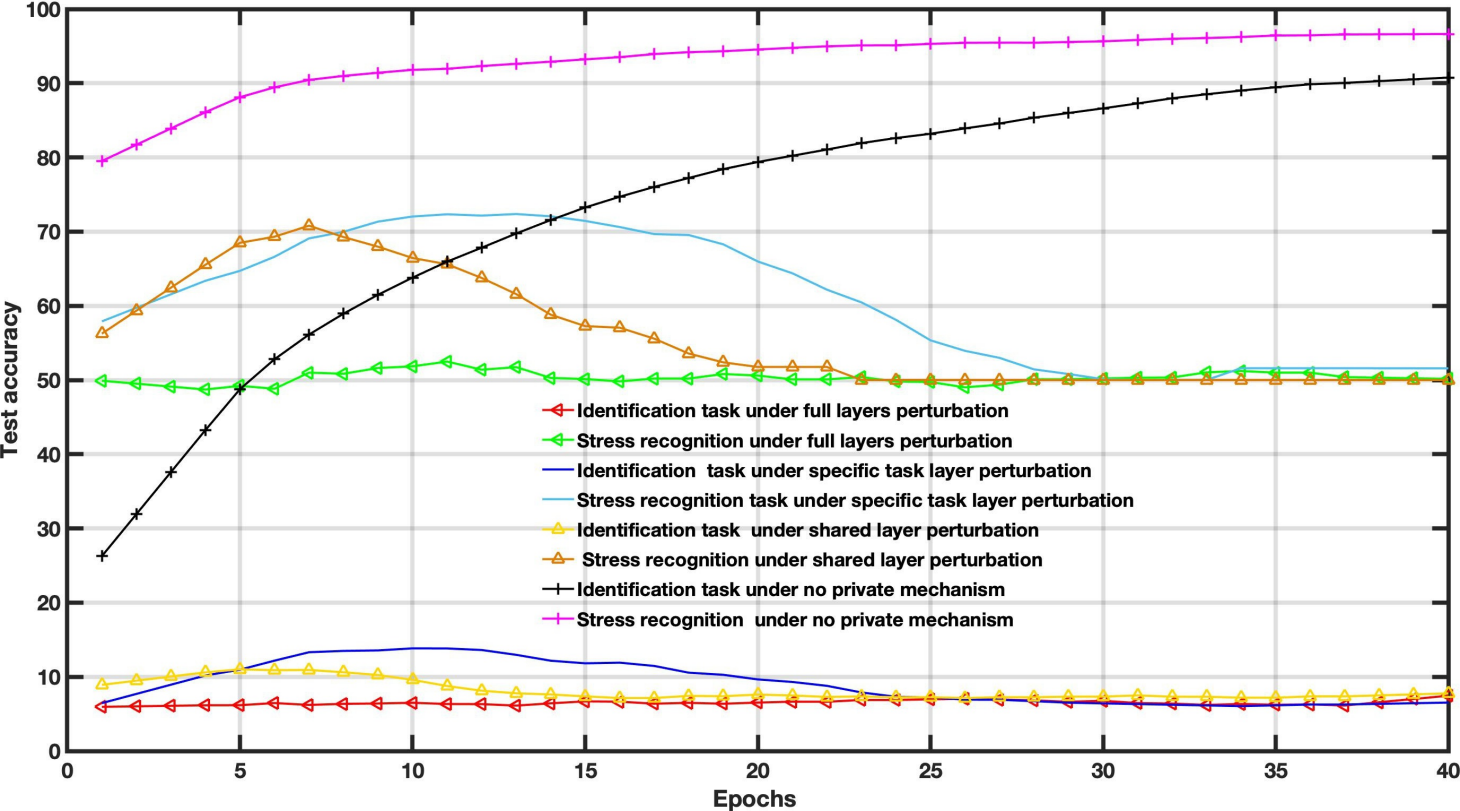
Multitask federated learning performance under the differential privacy mechanism “different layer perturbations” (wearable stress and affect detection, *ϵ*=5).

**Figure 10.**
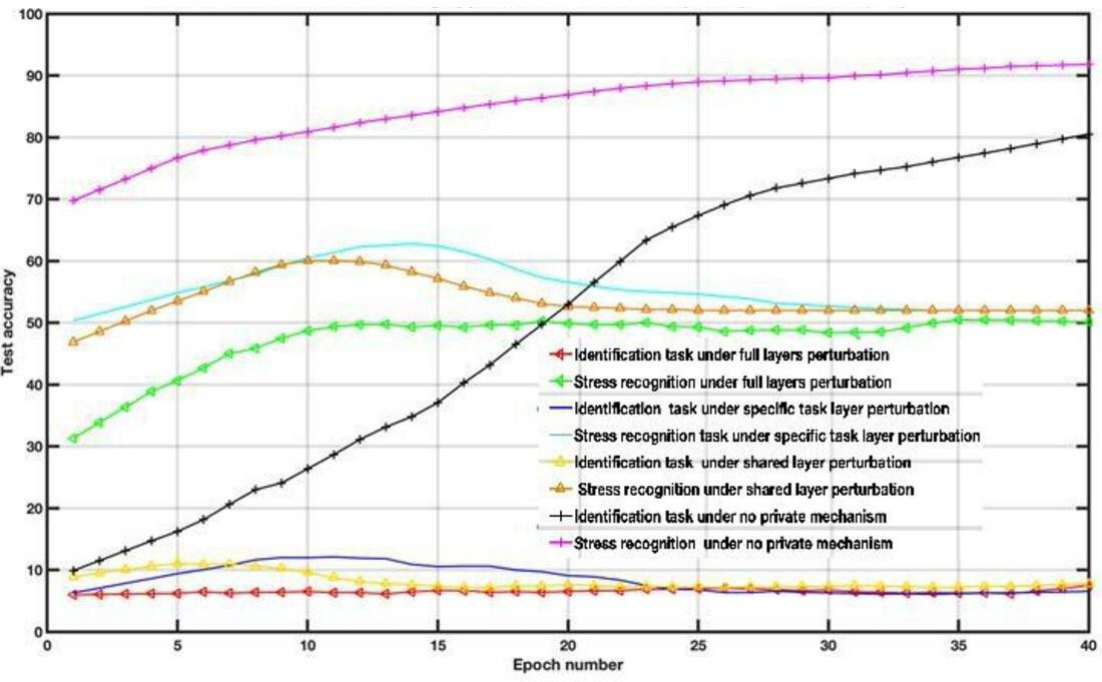
Multitask federated learning performance under the differential privacy mechanism “different layer perturbations” (virtual environment for real-time biometric interaction and observation, *ϵ*=5).

**Figure 11.**
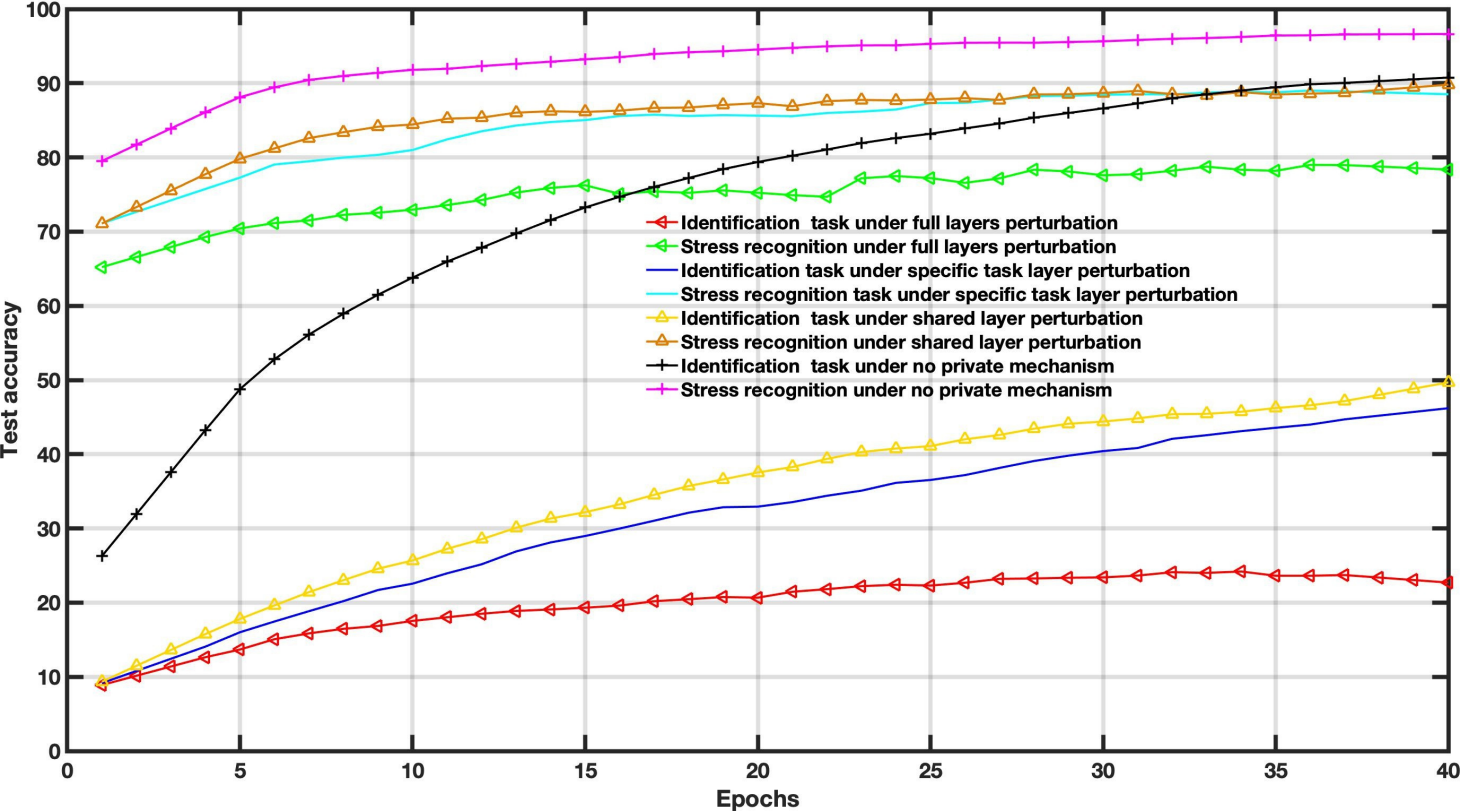
Multitask federated learning performance under the differential privacy mechanism “different layer perturbations” (wearable stress and affect detection, *ϵ*=15).

**Figure 12.**
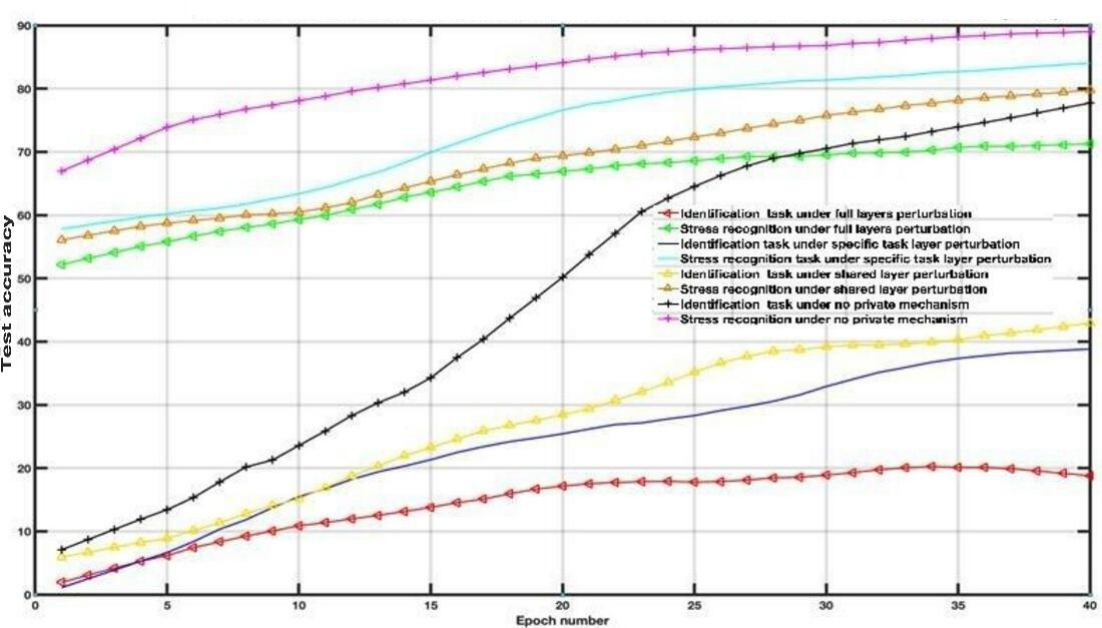
Multitask federated learning performance under the differential privacy mechanism “different layer perturbations” (virtual environment for real-time biometric interaction and observation, *ϵ*=15).

**Figure 13.**
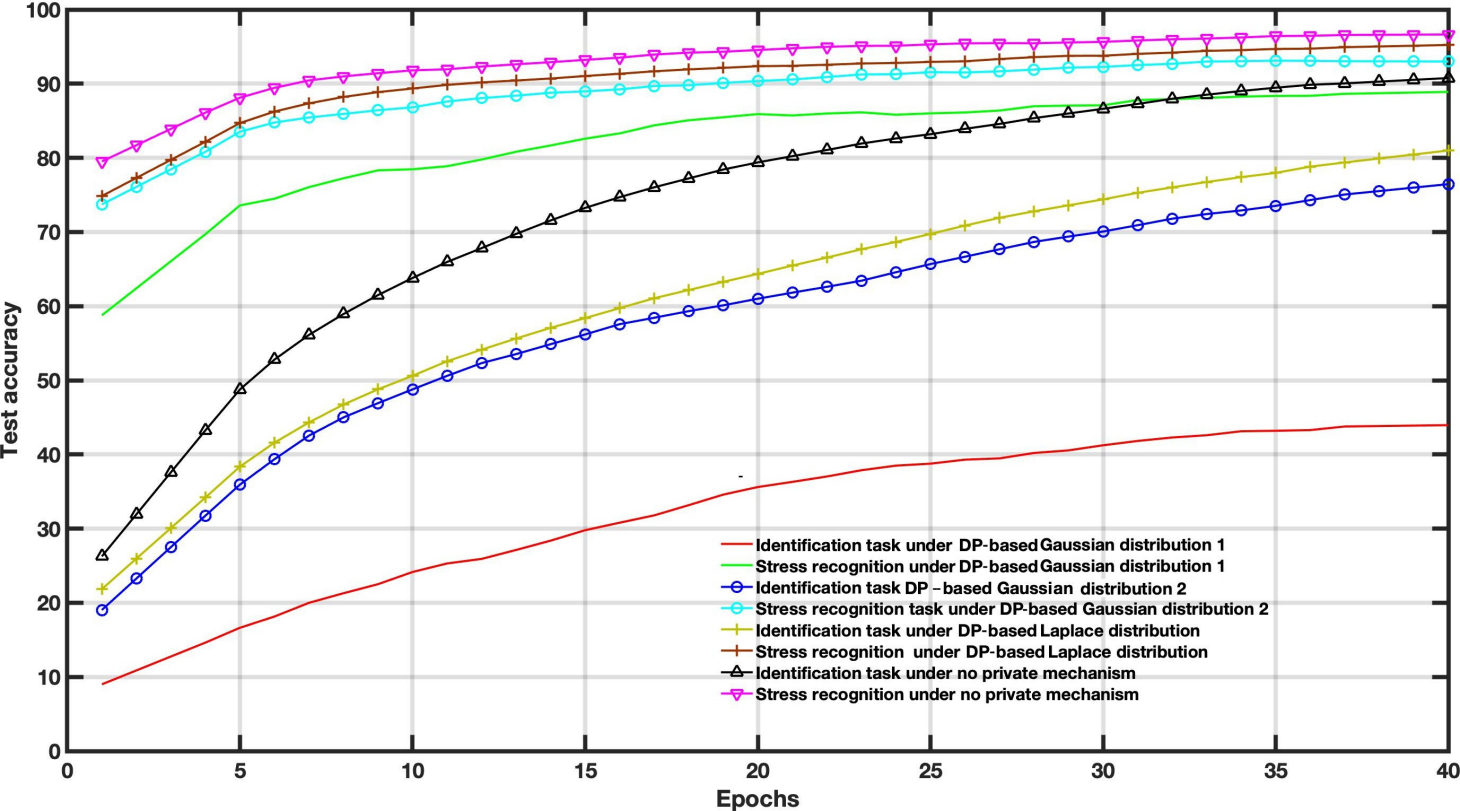
The evaluation results of the multitask federated learning approach with 3 differential privacy (DP) distributions (various layer task perturbation, *ϵ*=15).

**Figure 14.**
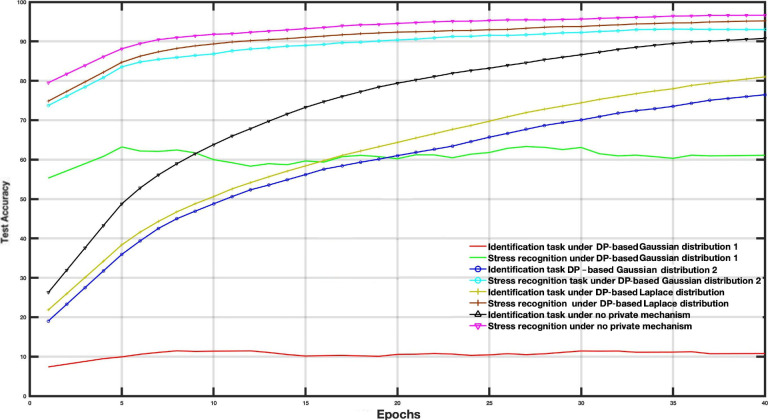
The evaluation results of the multitask federated learning approach with 3 differential privacy (DP) distributions (full perturbation, *ϵ*=15).

To examine the DP impact on the utility-privacy trade-off, we assessed the performance of MFL with a DP mechanism in WESAD and VERBIO datasets under the aforementioned scenarios. The DP budgets are set as follows *ϵ*=1, *ϵ*=15, *ϵ*=50 and *ϵ*=2000 for this experiment. As depicted in Figures 7-14 compared to the baseline scenario, that is, no privacy enhancing mechanism, adding DP noise into both shared and specific layers provides better results for utility performance; however, in terms of privacy, the perturbing specific task layer scheme provides better results than the perturbing shared layer. Results show that FL with perturbing all layers slows the convergence compared to others, although it provides better privacy (ie, decreasing identification accuracy).

Intuitively, our results demonstrate that adding noise to upper layers (identity recognition layers) effectively achieves a better privacy-utility trade-off. This advantage comes at the expense of a formal quantification of the relationship between learning features, that is, what we aim to share, and private variables, that is, what we aim to protect, which is rarely available in practice. We also evaluate the impact of noise distribution type on the proposed framework performance. The results from the WESAD dataset demonstrate that adding Laplace noise in our local training model preserves the stress recognition accuracy better than the Gaussian noise types (Figures13  14). However, it also maintains the identity recognition task performance.

Nevertheless, using the Gaussian mechanism (ie, Gaussian 2) increases the privacy level of the local training model because increasing the number of global iterations will also negatively affect its global convergence performance, that is, a larger T would increase the noise level variance, dramatically decreasing the global accuracy model (equation 13). In addition, we have found that a larger K contributes to avoiding the vanishing local SGD gradient problem; however, a larger *N* leads to a scale-down in the variance of injected noise level to model parameters and fools the SGD training inference.

When DP is also used in FL settings, our experiments suggest that it achieves encouraging performance even on lower budget values (ie, increasing privacy requirements). The compromise of physiological data privacy can have significant consequences for a life of individuals. It provides an opening for data attackers and puts them at danger of data breaches, which can result in several threats [35]. For instance, Shen et al [36] provided an analytic study to estimate the leakage level of the global training FL model about user’s information, and they generated a membership attack system on the global model to check if the target user is participated in training FL model or not. The experiences have been validated and tested on health data, called the University of Michigan Intern Health Study. The authors suggested to use DP to protect user-level privacy while maintaining the utility task performance. In similar work [37], applied personalized FL in recognizing stress using mobile health data (ie, WESAD) to decrease the information leakage from the global FL model.

Besides, there are also other risks including the possibility of revealing a soft biometric (eg, gender, location, and authenticity) and a hard biometric (ie, identity). So far, most of the research in using physiological modalities in both biometric domains focused on ECG, electroencephalography, EDA, and photoplethysmography [35,38]. We found that the model obviously achieves a better balance between the stress and identification classification task when multimodal signals are combined and used as an input compared to the single modality. However, as aimed in our work, it is difficult to provide privacy guarantees since there is no study that provides an investigation on which modality can reveal sensitive information about the subject. In this experiment, we provide a comparative study among these used modalities for both tasks, subject identification and emotion recognition. According to Tables3  4, we found that the ECG and acceleration modalities always led to an increase in the performance of the identification task. This fact might have contributed to their popularity in the biometric field [38]. This observation hints us that discarding model parameters containing sensitive information learned from modalities associated with identity information might help to effectively achieve a good privacy-utility trade-off to a certain extent, that is, decrease information leakage. Although for these modalities (eg, ACC and ECG for WESAD), the identification accuracy is higher than binary stress recognition accuracy, in general, it is lower than stress recognition performance. We can interpret this fact by the number of labels and the complexity of the task. For WESAD, there are 15 participants and for VERBIO, there are 55 participants, which corresponds to 15 and 55 different labels for biometric identification ML systems. If we think that the signals of some people can be similar under the same experimental conditions, it can cause some confusion for the ML algorithm and can cause some performance decrease. On the other hand, for stress, we used binary labels, and although the stress response can change from individual to individual, it can be stated that it is a relatively simpler task with 2 labels.

**Table 3.** The effect of modality on the stress and subject identification performance (wearable stress and affect detection).

Modalities and task		Approach								
		Centralized			FL^a^			FL+DP^b^		
		ACC^c^	*F*_1_	AUC^d^	ACC	*F*_1_	AUC	ACC	*F*_1_	AUC
**ACC**										
	Stress	91.60	86.09	92.01	89.90	84.81	90.35	84.46	79.23	85.00
	Subject identification	95.50	91.70	96.00	94.88	90.76	96.34	58.19	52.01	60.00
**BVP^e^**										
	Stress	98.34	96.14	99.13	96.05	92.01	97.00	90.97	86.92	92.46
	Subject identification	94.96	90.88	95.00	92.05	88.03	92.88	54.95	49.89	55.00
**EDA^f^**										
	Stress	99.00	98.01	99.00	95.29	91.09	95.57	91.27	86.03	92.00
	Subject identification	86.15	70.06	86.88	85.89	80.78	86.27	50.05	43.00	50.05
**ECG^g^**										
	Stress	97.01	95.00	97.02	93.37	89.03	94.00	91.60	86.45	92.10
	Subject identification	98.62	96.54	99.00	90.96	82.89	91.50	68.20	61.03	69.00
**TEMP^h^**										
	Stress	99.00	98.00	99.00	94.88	90.76	95.23	90.00	85.00	90.00
	Subject identification	97.88	95.78	98.00	91.22	86.01	92.00	51.23	43.00	52.00
**All**										
	Stress	99.86	96.72	99.86	95.56	91.38	96.13	90.00	85.00	90.00
	Subject identification	98.56	96.47	99.00	92.00	88.00	92.00	47.00	40.00	47.00

a FL: federated learning.

b DP: differential privacy.

c ACC: acceleration.

d AUC: area under the curve.

e BVP: blood volume pulse.

f EDA: electrodermal activity.

g ECG: electrocardiogram.

h TEMP: skin temperature.

**Table 4.** The effect of modality on the stress and subject identification performance (virtual environment for real-time biometric interaction and observation).

Modalities and task		Approach								
		Centralized			FL^a^			FL+DP^b^		
		ACC^c^	*F*_1_	AUC^d^	ACC	*F*_1_	AUC	ACC	*F*_1_	AUC
**ACC**										
	Stress	90.61	82.56	91.50	85.48	80.13	86.45	82.18	77.02	83.03
	Subject Identification	86.42	83.00	87.12	84.40	79.16	85.00	63.60	58.46	63.60
**BVP^e^**										
	Stress	92.96	88.87	93.00	88.95	83.84	90.56	85.00	80.00	85.00
	Subject Identification	85.63	80.31	86.33	80.86	74.37	90.13	48.64	42.51	49.00
**EDA^f^**										
	Stress	93.16	89.01	94.56	92.46	88.23	93.00	89.24	84.06	90.01
	Subject identification	88.30	83.12	88.30	83.83	78.65	84.23	45.12	38.01	46.00
**TEMP^g^**										
	Stress	92.64	88.37	93.34	90.42	85.12	90.42	86.32	81.12	87.06
	Subject identification	85.34	80.07	86.17	82.56	76.33	82.56	39.59	31.42	40.00
**All**										
	Stress	95.00	91.01	95.00	93.96	89.88	94.00	90.94	85.89	90.94
	Subject Identification	90.00	85.01	90.00	88.92	83.84	89.10	38.46	30.18	39.35

a FL: federated learning.

b DP: differential privacy.

c ACC: acceleration.

d AUC: area under the curve.

e BVP: blood volume pulse.

f EDA: electrodermal activity.

g TEMP: skin temperature.

### Comparison With State of the Art

The proposed approach is also compared with existing approaches used for stress recognition in FL settings, and the results are reported in Table 5. Most similar works have mainly focused on the benefits of FL in affective computing by generating data clients from unique datasets (ie, as used in the FL framework with standard benchmarks, namely Modified National Institute of Standards and Technology, Canadian Institute for Advanced Research-10, and Canadian Institute for Advanced Research-100). To the best of our knowledge, the number of studies using physiological signals in FL settings is relatively low compared to other case studies in affective computing, such as speech and facial expression–based studies. According to Table 5, we can see that existing works used only FedAvg with the WESAD dataset and achieved similar performance, as confirmed by our previous study. To the best of our knowledge, our paper both improves privacy protection and provides slightly higher accuracies when compared with similar studies. Consistent with compared works, for both databases, WESAD and VERBIO, the obtained performance behaves similarly.

**Table 5.** Comparison of affect recognition studies using federated learning (FL) and privacy-preserving approaches.^a^

Study	Dataset	FL algorithm	Data split	Accuracy
Almadhor et al [39]	WESAD^b^	FedAvg^c^+logistic regression	N/A^d^	86.82
Fauzi et al [40]	WESAD	FedAvg+DNN^e^ network	N/A	85.75
Can and Ersoy [15]	Private dataset	FedAvg+MLP^f^	N/A	88.55
Lee et al [29]	WESAD	FedAvg+MLP	LOOCV^g^	75.00
Our previous study [38]	WESAD	FedAvg+1 DCNN^h^+DP^i^	5-fold CV^j^	90.00
This study	VERBIO^k^	FedAvg+1 DCNN+DP	5-fold CV	88.67

a The table reports the main approaches have been applied for stress detection by using a physiological dataset.

b WESAD: wearable stress and affect detection.

c FedAvg: federated averaging.

d N/A: not applicable.

e DNN: deep neural network.

f MLP: multilayer perceptron.

g LOOCV: leave-one-out cross-validation.

h DCNN: deep convolutional neural network.

i DP: differential privacy.

j CV: cross-validation.

k VERBIO: virtual environment for real-time biometric interaction and observation.

Unlike the compared works [15,29,38,40], which analyze privacy concerns in stress recognition, we introduced a multitask model enabling us to strike a balance between utility and privacy. This is achieved by selectively adding noise to layers prone to subject information leakage. The comparison provided in Table 5 reveals that prior studies achieved performance comparable to our case study when using FedAvg on WESAD or a private dataset. Through our experiments, we provided a comprehensive investigation of how to balance privacy preservation and stress recognition. The obtained results demonstrate that the combination of MFL and DP can maintain good performance without sacrificing the user’s privacy.

## Discussion

The overall objective of our work was to present an approach to stress recognition that ensures robustness while protecting the user’s privacy. To this end, we developed a personalized multitask federated model framework with DP. We used a user-level DP mechanism by injecting an amount of noise into personalized layers for perturbing identity while preserving task-specific utility. Using multitask and DP (*ε*=15), we obtained 90% and 87% accuracy for recognizing emotions while limiting the reidentification accuracies to 47% and 38% on WESAD and VERBIO, respectively. We extensively tested different parameters including layer of neural network, privacy budget, and different noise distributions. As expected, adding noise to the upper layer decreases the affect recognition performance less when compared to the last task-specific layers. We also demonstrated the effect of data distribution on the performance. Having said that, the paper is not without limitations. Although we tested our algorithms with state-of-the-art datasets (WESAD 15 participants and VERBIO 55 participants), especially for the biometric task, larger datasets with a more heterogenous and higher number of participants should be used. Furthermore, these datasets were recorded in a controlled environment. Real-life or in-the-wild datasets will create more challenges and might require more advanced and complex architectures. However, with the available data, we managed to develop an automatic stress recognition system with around 90% accuracy by keeping biometric identification accuracy below 40%. We believe that our results will help researchers in determining suitable parameters and distributions for achieving the desired trade-off between utility and privacy for affective computing applications. Currently, new gradient-based unsupervised adversarial attackers are attacking deep neural classification models to infer the privacy of the distributed training gradient. In future works, to address this threat, we are planning to conduct additional experiments with federated differentially private generative adversarial networks that can provide better privacy protection and data diversity for widespread applications of physiological computing systems.

## Supplementary material

10.2196/60003 Multimedia Appendix 1 Three different scenarios for adding noise with differential privacy: adding noise to only shared layers, task-specific layers, and full layers.

10.2196/60003 Multimedia Appendix 2 Stress versus identity recognition performance of multitask centralized learning using 2 different datasets.

## Abbreviations

CL: centralized learning
CNN: convolutional neural network
DP: differential privacy
ECG: electrocardiogram
EDA: electrodermal activity
FedAvg: federated averaging
FL: federated learning
IID: independent and identically distributed
MFL: multitask federated learning
ML: machine learning
MTL: multitask learning
SGD: stochastic gradient descent
VERBIO: virtual environment for real-time biometric interaction and observation
WESAD: wearable stress and affect detection
