# Supplementary figures and images for: Balancing Between Privacy and Utility for Affect Recognition Using Multitask Learning in Differential Privacy–Added Federated Learning Settings: Quantitative Study
Source: JMIR Ment Health. 2024 Dec 23;11:e60003. doi: 10.2196/60003 (PMC11684349; doi:10.2196/60003)

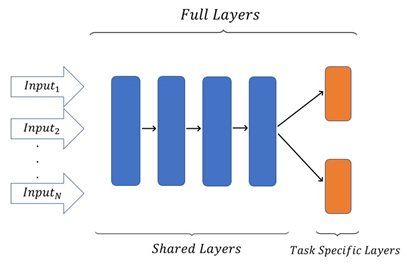

Supplement: Multimedia Appendix 1 [file mental-v11-e60003-s001.png]

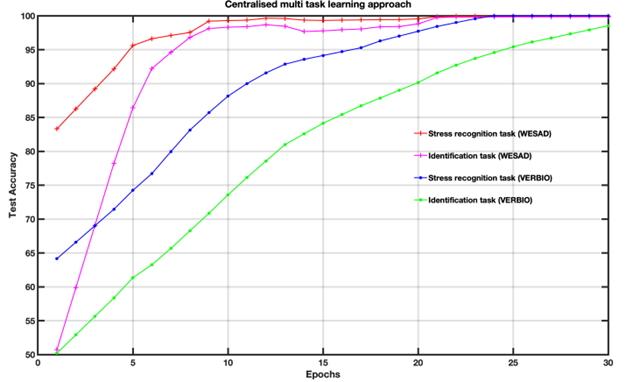

Supplement: Multimedia Appendix 2 [file mental-v11-e60003-s002.png]
